# Designing a culturally responsive mindfulness-based blood pressure program: the IndigenousMIND/MB-BP protocol

**DOI:** 10.3389/fpubh.2026.1911281

**Published:** 2026-10-21

**Authors:** Jeffrey Proulx, Chase M. Bryer, Sherente Harris, Dawn Dove, Marinna Okawa, Lynn Koerbel, Erin Woo, Lila Chamlagai, Dikchit Acharya, Eric B. Loucks

**Affiliations:** 1 Department of Behavioral and Social Sciences, Brown University School of Public Health, Providence, RI, United States; 2 Mindfulness Center at Brown University, Providence, RI, United States; 3 Narragansett Tribal Community, Narragansett Tribe Rhode Island, RI, United States; 4 Department of American Studies, Brown University, Providence, RI, United States; 5 Warren Alpert Medical School of Brown University, Providence, RI, United States; 6 School of Professional Studies, Brown University, Providence, RI, United States; 7 Department of Epidemiology, Brown University School of Public Health, Providence, RI, United States

**Keywords:** culture, indigenous knowledge, mindfulness, protocol and guidelines, wellness

## Abstract

**Introduction:**

Indigenous cultures across Turtle Island have long promoted wellness through language, ceremony, storytelling, movement, community connection, and relationships with the natural world. These cultural practices may serve as important pathways for healing and health promotion. At the same time, Indigenous communities experience disproportionate rates of cardiovascular disease (CVD), with hypertension representing a major modifiable risk factor. Although Mindfulness-Based Blood Pressure Reduction (MB-BP) has demonstrated clinically meaningful reductions in blood pressure, standard mindfulness interventions often lack the cultural grounding necessary for sustained engagement in Indigenous communities. This study describes the development and pilot implementation of an Indigenous-focused mindfulness program (IndigenousMIND) with the MB-BP class, a culturally grounded adaptation that integrates MB-BP with Indigenous language, cultural teachings, ceremony, and community-defined contemplative practices.

**Methods:**

Thirteen Indigenous adults participated in an eight-week IndigenousMIND/MB-BP program. Using a rapid-cycle adaptation process, we approached MB-BP with culturally adapted programming in conjunction with tribal cultural leaders, and delivered in a tribally specific format. Adaptations incorporated tribal language, storytelling, ceremonial elements, ecological relationships, and relational learning practices while preserving MB-BP’s core mechanisms. Qualitative reflections collected at the final community gathering were analyzed thematically to assess feasibility, acceptability, and perceived impact. The adaptation process also produced a structured IndigenousMIND/MB-BP protocol and facilitator guide.

**Results:**

Participants reported high engagement and strong acceptability. Five primary themes emerged: (1) connection and community, (2) cultural identity and preservation, (3) mindfulness and personal transformation, (4) intergenerational healing and responsibility, and (5) challenges and resilience. Participants described mindfulness as consistent with Indigenous ways of knowing and identified language, storytelling, ceremony, and connection to land as meaningful pathways into practice. Reflections emphasized cultural reconnection, community belonging, and ecological awareness as important components of healing and well-being.

**Discussion:**

IndigenousMIND/MB-BP demonstrates the feasibility of integrating evidence-based hypertension prevention with Indigenous cultural strengths and community leadership. The resulting protocol provides a model for community-led adaptation of mindfulness-based health interventions and offers a framework for future studies addressing cardiovascular health equity in Indigenous communities.

## Introduction

A consistent finding across behavioral medicine, public health, and implementation science is that interventions are most likely to be effective and sustained when they are designed to fit the cultural contexts, relational structures, and lived realities of the communities they intend to serve (1–3). Culture shapes how health is defined, how suffering is interpreted, how healing is sought, and what kinds of practices feel legitimate and safe (4). When interventions are culturally incongruent—however “evidence-based” they may be in other settings—adoption, retention, and long-term sustainability often falter, particularly in communities that have experienced systemic marginalization or medical exploitation (5, 6). In the public health literature, frameworks for cultural adaptation emphasize that cultural responsiveness should not be superficial (e.g., imagery or translation alone), but should also reflect deeper structures such as values, social norms, meaning systems, and community priorities (7, 8). Within Indigenous health sciences, the rationale is often even stronger: cultural continuity is not simply an engagement strategy, but a central determinant of wellness, identity development, and community resilience, and therefore a plausible mechanism of change in many forms of healing work (4).

Recent scholarship across culturally and socially diverse populations has further emphasized that effective health promotion requires attention not only to individual behavior, but also to the cultural, relational, spiritual, and community systems through which health and well-being are understood. For example, emerging frameworks in Middle Eastern and Asian contexts have argued that mental health interventions may be strengthened when they are embedded within local cultural values, social relationships, community structures, and meaning systems rather than simply translating or modifying interventions developed elsewhere (9–12). Related intervention research suggests that mindfulness practices can improve psychological outcomes when adapted to the developmental and social contexts of the populations being served (13). Although these studies arise from populations and health concerns distinct from those addressed here, collectively they reflect a broader movement away from assumptions that evidence-based interventions can be transferred unchanged across cultural contexts.

This broader literature converges with, but does not substitute for, a more specific body of Indigenous health scholarship in which culture may function as more than a means of increasing intervention acceptability. Indigenous scholars and communities have long advanced approaches in which cultural connection, spirituality, language, community relationships, and intergenerational transmission are themselves understood as resources for healing and resilience. Recent work on multigenerational resilience similarly emphasizes the potential role of culture and spirituality in transmitting resilience across generations in Indigenous communities (14). A systematic scoping review of culturally grounded therapeutic approaches across Indigenous populations likewise found that culturally based services may support healing and well-being by strengthening cultural connection, safety, empowerment, and social and spiritual support (15). Within mindfulness specifically, culturally adapted mindfulness-based interventions for First Nations peoples have demonstrated feasibility and acceptability, with cultural and social identity emerging as important dimensions of participants’ experiences (16).

Importantly, Indigenous scholarship suggests that cultural responsiveness can extend beyond adapting conventional interventions to better fit Indigenous participants. Wendt et al. (17) distinguish culturally adapted treatment from Indigenous grassroots and traditional healing approaches and argue for greater attention to “culture-as-treatment,” in which Indigenous cultural practices and systems of meaning are themselves recognized as therapeutic resources. This distinction is particularly relevant to the present study. Whereas the broader cultural-adaptation literature establishes the importance of situating interventions within local systems of meaning, Indigenous scholarship suggests a stronger proposition: culture need not simply be adapted into an intervention; cultural practices, relationships, language, spirituality, and connections to land and community may themselves constitute pathways through which healing, self-regulation, identity, and well-being are supported (15, 17). This perspective provides an important foundation for an adapted program for Indigenous communities, which seeks not simply to modify the presentation of an established mindfulness intervention, but to embed its evidence-based functions within Indigenous cultural practices and relationships that may themselves contribute to health and healing.

For Indigenous Peoples on Turtle Island, the need for culturally grounded intervention design cannot be separated from a history in which colonization deliberately targeted and disrupted the very practices that supported collective regulation, identity continuity, and relational accountability: language, ceremony, land-based lifeways, kinship systems, and community governance (18). These disruptions are widely recognized as foundational drivers of social and health inequities and remain actively reproduced through structural conditions that constrain access to land, resources, culturally safe care, and self-determination (19). In this context, the “fit” of a health intervention is not a technical matter—it is historical and relational. Effective interventions must do more than deliver health information; they must support healing in ways that restore relationships to culture, to land, to ancestors, and to one another, while also providing practical tools for addressing chronic disease risk. Indigenous theories of stress and healing argue that cultural revitalization and relational accountability can function as protective factors against stress-related morbidity, particularly when culture is enacted as practice rather than treated as symbolic context (4, 20).

Due at least in part to the ongoing impacts of racism and colonization, cardiovascular disease (CVD) remains a major driver of morbidity and premature mortality across Indigenous communities within North America (21). Hypertension is among the most important modifiable contributors to CVD risk and intersects with high prevalence of diabetes, chronic kidney disease, and stroke. Hypertension is also a plausible physiological pathway through which chronic stress, trauma exposure, sleep disruption, and structural adversity can contribute to cardiometabolic disease, making it a particularly relevant target for interventions that combine behavioral, psychosocial, and cultural pathways.

Epidemiological studies across time underscore both the magnitude and persistence of hypertension in Indigenous communities. The Strong Heart Study, one of the most influential studies in Indigenous cardiovascular epidemiology, documented wide variability in hypertension prevalence across participating tribal communities—ranging from approximately 27–56%—and also revealed that, even with moderate rates of awareness and treatment, blood pressure control remained low for many participants (22). More recent cohort work indicates that the burden remains severe, particularly among Indigenous elders. For example, analyses of American Indian and Alaska Native Medicare beneficiaries highlight a high burden of cardiovascular conditions and cardiometabolic risk factors, including hypertension affecting roughly 70–75% of individuals 5–15% higher prevalence than non-Hispanic White beneficiaries reinforcing the need for scalable strategies to address cardiovascular inequities in later life. From a public health standpoint, this matters because even modest reductions in systolic blood pressure translate into meaningful reductions in cardiovascular events at the population level (23). A large meta-analysis of blood pressure-lowering randomized controlled trials found that reducing systolic blood pressure by 5 mmHg is associated with about a 10% reduction in major cardiovascular events, including myocardial infarction and stroke, supporting the clinical and public health relevance of interventions that can achieve modest but durable reductions (24).

While hypertension is biologically modifiable, the real-world conditions that determine whether a program is accepted and sustained are often social, historical, and relational. Indigenous communities have repeatedly experienced substandard care, dismissive encounters, and ongoing structural discrimination within health systems, which can reasonably lead to avoidance of preventive services and lower trust in externally developed interventions. In Canadian contexts, qualitative work with providers and decision-makers has documented discrimination against Indigenous patients as a persistent barrier to equitable care and an obstacle to effective engagement (25). In U.S. contexts, survey-based research has documented substantial proportions of Native American respondents reporting discrimination in a range of institutional settings, including medical contexts, and such experiences are consistently linked with poorer self-reported health and reduced engagement (26). These experiences plausibly reduce uptake of prevention and follow-up services—precisely the activities needed for effective hypertension prevention and management.

In addition to relational barriers, there are persistent knowledge gaps that may reflect uneven access to culturally appropriate cardiovascular education and prevention programming. Studies have documented disparities in knowledge of heart attack and stroke symptoms among Indigenous adults, which can contribute to delays in care and worsen outcomes (27). In urban Indian health contexts, targeted health promotion programs have demonstrated that cardiovascular knowledge can be improved through culturally adapted curricula, supporting the value of culturally tailored education as part of broader prevention strategies (28). Collectively, these findings highlight a pattern: Indigenous communities face high cardiovascular burden, and yet the systems designed to reduce that burden often fail to engage in ways that are culturally resonant, historically informed, and locally sustainable. This gap creates a strong rationale for interventions that center Indigenous cultural strengths, increase self-regulation capacities, and rebuild trust through relationally accountable partnership.

A number of cardiovascular initiatives have recognized the need for cultural responsiveness, yet many still struggle to offer clear, replicable strategies for integrating culture into the intervention as the core delivery mechanism. For example, frameworks such as the American Heart Association’s “Life’s Essential 8” provide a strong behavioral and risk-factor target structure, but do not inherently solve the “how” of culturally grounded delivery in communities shaped by historical trauma and cultural suppression (29). Some Indigenous-focused initiatives emphasize stakeholder input and inclusion of traditional healers; however, the degree to which culture is embedded as a lived, experiential, weekly practice varies considerably. In many cases, culture is treated as a contextual feature rather than as a mechanism of healing that participants can embody across time.

Mindfulness-based interventions have increasingly been studied as platforms for health behavior change, stress reduction, and cardiometabolic outcomes (30). Mechanistically, mindfulness training is theorized to improve self-regulation capacities—attention control, emotion regulation, and self-awareness—processes that are directly relevant to hypertension determinants such as stress reactivity, dietary patterns, physical activity, sleep, and substance use. Mindfulness training also supports interoceptive awareness (awareness of internal bodily sensations), which is increasingly recognized as central to self-regulation and health behavior adherence (31). Mindfulness-based interventions have demonstrated effects on stress, depression, and anxiety symptoms across populations, and these improvements may support downstream physiological outcomes through reduced sympathetic arousal and improved coping (32). However, for Indigenous communities, mindfulness must be approached carefully. Many mainstream mindfulness programs are culturally “neutral” in presentation, and cultural neutrality can function as cultural erasure when delivered in communities where language loss, ceremony disruption, and historical trauma are central (33). For this reason, Indigenous-led mindfulness adaptation work increasingly emphasizes that contemplative practice must be relational, place-based, and culturally specific, honoring local knowledge systems and restoring practices that colonization attempted to suppress (16, 33).

Mindfulness-Based Blood Pressure Reduction (MB-BP) is an 8-week program designed explicitly to target hypertension determinants and reduce blood pressure through mindfulness training combined with education and behavioral support. MB-BP adapts core mindfulness training principles and structures them around cardiovascular risk reduction, including stress reactivity, diet, physical activity, and medication adherence. The MB-BP randomized clinical trial demonstrated clinically meaningful reductions in systolic blood pressure compared with enhanced usual care (34). Earlier trial work established feasibility and acceptability of MB-BP and documented reductions in blood pressure consistent with clinically meaningful effect sizes (35). Mechanistic work within MB-BP has emphasized interoceptive awareness as a pathway for improved adherence to dietary recommendations such as DASH, supporting the conceptual model that mindfulness changes key self-regulation processes relevant to hypertension management (31).

Although MB-BP offers a strong evidence base, it was not designed specifically for Indigenous communities, and therefore does not inherently incorporate Indigenous language, cultural teachings, ceremony, or tribal governance structures. This creates an important opportunity: rather than inventing a new hypertension intervention from scratch, it may be possible—and preferable—to culturally adapt a rigorously tested program while maintaining its mechanisms and educational targets. Doing so requires a thoughtful adaptation process—one that preserves core components, respects Indigenous sovereignty and ethics, and produces a protocol that can be sustained by community members after the research period ends.

The IndigenousMIND (36) framework emerged from long-term partnerships across Indigenous communities and was developed as a culturally grounded mindfulness intervention development approach. Rather than treating mindfulness as a standalone technique delivered to participants, IndigenousMIND treats culture itself as the primary vehicle for mindful practice and healing. In this model, mindfulness is cultivated through culturally meaningful practices that already embody focused attention, reverence, reciprocity, and relational accountability—such as storytelling, dance, beading, prayer, language learning, and land-based practices. These practices are not “added” to a mindfulness program; they are the foundation through which mindful awareness is embodied.

A crucial element of IndigenousMIND is sustainability through capacity building. The program is designed so that community members—elders, culture-bearers, clinicians, educators, and youth leaders—can become facilitators and stewards, thereby reducing dependence on external researchers. The program development process prioritizes long-term relationship, iterative co-design, and the return of value to communities through training, paid roles, and usable products (e.g., recordings, curricula, and facilitation tools). In this way, IndigenousMIND aligns with Indigenous health governance principles emphasizing reciprocity, community benefit, and relational accountability.

### Objective

The present project integrates IndigenousMIND with MB-BP to form IndigenousMIND/MB-BP, an 8-week program intended to address hypertension risk through the proven structure and mechanisms of MB-BP while embedding Indigenous language, cultural teachings, and culturally meaningful mindful practices through the IndigenousMIND framework. The integration was intentionally designed and led by the primary developers of both parent interventions—Dr. Eric Loucks (MB-BP) and Dr. Jeffrey Proulx (IndigenousMIND)—allowing adaptation decisions that maintained fidelity to MB-BP’s core components while creating cultural immersion consistent with IndigenousMIND.

The pilot context for this integration was a Northeast Tribal community, where prior mindfulness initiatives and culturally grounded intergenerational programming demonstrated strong community interest and engagement. In preparation for the hypertension-focused program, the community had already engaged in culturally immersive mindfulness activities such as intergenerational mindful crafting, where elders and younger tribal members practiced meditation and then engaged in cultural arts guided by prayer, song, and tribal teachings. These prior experiences offered both relational foundation and proof-of-concept that a culturally immersive mindfulness approach could be acceptable and highly engaging.

In this manuscript, we describe the development and pilot implementation of IndigenousMIND/MB-BP as a protocol development effort. We emphasize the adaptation process, session structure, and real-time development pipeline that produced an integrated handbook and protocol intended for future replication and adaptation. Consistent with integrative medicine values, the program is designed to address not only physiological risk factors but also the emotional, relational, and spiritual dimensions of wellness, recognizing that hypertension risk in Indigenous communities is entangled with stress physiology, historical trauma, and culturally mediated pathways of healing.

## Methods

This project was conducted as a community-engaged pilot implementation focused on feasibility and structured protocol development of the IndigenousMIND/MB-BP intervention within a Northeast Tribal community. The primary purpose of this manuscript is to document the development process and resulting protocol rather than to report definitive efficacy outcomes. The study included recruitment of a small cohort, delivery of an 8-week group program, collection of feasibility indicators (e.g., recruitment pathways, attendance), and iterative documentation of session adaptations that culminated in an integrated curriculum and handbook for future use.

The intervention was implemented in partnership with a Northeast Tribal community. The project built upon a multi-year relationship between the investigative team and the community, including prior culturally grounded mindfulness programs and participation of community members in broader Indigenous mindfulness networks. The partnership emphasized relational accountability, respect for tribal governance, and commitment to developing programming that could be sustained locally beyond the research period. Importantly, the present intervention was not the research team’s first mindfulness program developed with this Tribal community. The pilot emerged from several years of community-engaged programming that included multi-week mindfulness courses, weekend retreats, intergenerational mindfulness activities incorporating cultural arts and shared food, and programs integrating mindfulness with movement, yoga, and dance. Community members and cultural leaders participated in the development and delivery of these activities across this period, allowing the approach to be iteratively refined before the present pilot. Thus, the high level of acceptability observed in the present study should be understood in the context of a longstanding community partnership and an intervention-development process that had already incorporated substantial community guidance regarding culturally appropriate content, format, and delivery.

Eligible participants were adults aged 18 years or older who were members of the Tribal community. The 13 enrolled participants (10 women and 3 men) ranged in age from 47 to 72 years. Eligibility did not require a formal diagnosis of hypertension or elevated blood pressure. This decision was intentional and aligned with prevention-oriented public health approaches and Indigenous wellness perspectives that emphasize whole-person balance rather than disease-only inclusion criteria. Participants were able to enroll regardless of baseline blood pressure status, allowing the program to serve as both prevention and support for individuals with existing cardiovascular risk; mindfulness can serve primary, secondary, and tertiary aims of public health, from prevention to supporting those living with chronic conditions. Attendance was high across the eight-week intervention. Ten of the 13 participants attended all nine sessions (including an orientation), and the remaining three participants each attended six of the eight sessions. Overall, participants attended 98 of 104 possible participant-sessions (94.2%).

Participants were recruited through community-based channels, primarily word of mouth and informal dissemination through existing community networks. Recruitment leveraged relationships and community trust developed through prior programming. A total of 13 adults enrolled. Recruitment was not intended to produce a probability or demographically representative sample of the Tribe. Consistent with the protocol-development purpose of this pilot, we recruited a community-based convenience sample of adult Tribal members interested in participating in the eight-week program. Accordingly, participants should not be interpreted as statistically representative of the broader Tribal population.

IndigenousMIND/MB-BP was developed through direct collaboration between the originators of both parent interventions: Mindfulness-Based Blood Pressure Reduction (MB-BP; Dr. Eric Loucks) and IndigenousMIND (Dr. Jeffrey Proulx). This structure ensured that adaptation decisions preserved core MB-BP mechanisms while enabling cultural immersion consistent with IndigenousMIND. Delivery of the intervention within the Tribal community was led by three people: Dr. Proulx, along with a Tribal elder and a spiritual leader in the community, with ongoing consultation with Dr. Loucks to ensure alignment with the MB-BP structure.

A distinctive feature of this pilot was its real-time adaptation structure, designed to generate a complete integrated handbook and protocol across the 8-week intervention period. An MB-BP course led by Dr. Loucks was delivered weekly on Wednesdays. Dr. Proulx and the Tribal instructors attended the Wednesday sessions as participant-observers, taking structured notes on session flow, educational content, experiential practices, home practice assignments, and facilitation strategies. Attendance at the Wednesday sessions allowed the adaptation team to maintain fidelity to MB-BP sequencing and intent while gathering detailed information needed to translate session components into a culturally grounded form.

Following each Wednesday MB-BP session, Dr. Proulx and the Tribal instructors met on Fridays to adapt that week’s content for Saturday delivery. These meetings focused on identifying core MB-BP components that must remain intact (e.g., hypertension education targets, mindfulness mechanisms, home practice structure), selecting cultural elements (language, stories, metaphors, prayer, song, traditional understandings of breath and balance) that could serve as the vehicle for those components, and modifying session language, examples, and experiential practices to be culturally resonant and locally specific. The adapted session was then delivered to the Tribal cohort on Saturdays. This process was repeated for all eight sessions, resulting in a fully adapted handbook and protocol by the conclusion of the pilot.

This process also enabled systematic documentation of adaptations. Each week’s changes were recorded in a developing handbook, including adapted scripts, language insertions, culturally grounded metaphors, and revised practice instructions. When MB-BP introduced standard breath-focused (or alternative trauma-informed anchor) practices, the Tribal adaptation incorporated local linguistic and cultural framing of breath, including culturally meaningful concepts and language elements. These steps also included alternative anchors in a trauma sensitive manner (e.g., attention to palms of hand, soles of feet, sound). When MB-BP used nature-based guided imagery practices such as the Mountain Meditation, the adaptation retained the attentional and imagery structure while embedding tribal language for key elements and aligning the metaphor with Indigenous understandings of land, life cycles, and endurance. A similar approach was used for body scan practices, incorporating tribal language for body parts while retaining the interoceptive awareness goals central to MB-BP.

The IndigenousMIND/MB-BP intervention was delivered as eight weekly group sessions held on Saturdays. Sessions followed MB-BP’s general structure while embedding IndigenousMIND cultural immersion components. Each session typically included: (1) an opening (often incorporating prayer and/or song led by a community member), (2) a culturally adapted mindfulness practice, (3) MB-BP-aligned cardiovascular health education and discussion of hypertension determinants, (4) group reflection on practice, challenges to practice, insights and discoveries, and relational processing, and (5) assignment of home practices supported by adapted recordings and practice instructions.

Relationality was operationalized through the organization of the sessions as shared community practice rather than solely as individual skills training. Openings often included prayer and/or song led by community members; mindfulness practices were followed by collective reflection and relational processing; and Tribal language, stories, metaphors, and cultural teachings were used to connect individual awareness with relationships to family, community, ancestors, land, and the natural world. Tribal cultural leaders participated directly in both the adaptation and delivery of the intervention, reinforcing reciprocity, cultural continuity, and responsibility to community as part of the practice itself. In this sense, mindfulness was taught not only as a means of self-regulation, but as awareness enacted within relationships and responsibilities to others.

The intervention emphasized that mindfulness is not solely an internal cognitive skill but a relational practice grounded in respect, reciprocity, and connectedness. This framing aligned with IndigenousMIND’s approach and supported participant engagement by positioning contemplative practice as consistent with Indigenous ways of knowing and being, rather than as an imported method.

Given the protocol-development orientation, primary outcomes included feasibility indicators such as enrollment, attendance, and qualitative feedback regarding session relevance and acceptability. Blood pressure measurement was initially incorporated into the pilot as an exploratory feasibility measure. However, obtaining consistent participant-provided blood pressure measurements across the intervention proved difficult, resulting in insufficient paired blood pressure data for meaningful analysis. Blood pressure data collection was therefore discontinued during this protocol-development phase. Because the study was designed primarily to assess feasibility, cultural acceptability, and protocol development—and participants were not required to have hypertension or elevated blood pressure—blood pressure change was not evaluated as an efficacy outcome. This experience informed plans for subsequent efficacy testing, in which standardized blood pressure measurement and recruitment of participants with elevated blood pressure will be incorporated prospectively into the study design.

The IndigenousMIND/MB-BP curriculum and facilitator handbook were developed through an iterative, rapid-cycle adaptation process that paired (a) close observation of the parent MB-BP course with (b) structured weekly adaptation and documentation for delivery in a tribally specific IndigenousMIND context. This approach was designed to preserve MB-BP’s core intervention functions (e.g., self-regulation training, interoceptive awareness, health behavior linkage to blood pressure determinants) while intentionally embedding IndigenousMIND’s cultural–relational mechanisms (e.g., language, teaching stories, ceremonial framing, place-based metaphors) in ways that were acceptable, feasible, and meaningful for Tribal participants.

Each weekly cycle concluded with documentation updates to the emerging IndigenousMIND/MB-BP protocol (session plans, handouts, suggested language, and adaptation notes). Version control and change logs were maintained so that modifications across weeks were transparent and reproducible, consistent with recommendations that adaptation processes be explicitly documented to support future implementation and evaluation (37, 38). In keeping with “deep structure” cultural adaptation principles, cultural integration prioritized meaning, identity, and relational context—not merely surface-level tailoring (8). The process also aligned with community-based participatory research (CBPR) priorities emphasizing shared learning, co-ownership, and practical utility for community use beyond the research setting (39).

To assess participant experiences and to inform refinement of the curriculum, qualitative questions were administered at the final gathering following completion of the eight-week course. This gathering included shared food and an invited group discussion reflecting on the program. Participants provided brief written and/or oral reflections in response to open-ended prompts focused on perceived benefits, meaningful program elements, cultural resonance, barriers to participation, and suggestions for improvement. Responses were analyzed using reflexive thematic analysis (RTA) (40), emphasizing patterns of meaning relevant to feasibility, acceptability, and mechanisms of change.

Participants were invited to respond to a brief set of open-ended qualitative prompts focused on perceived benefits, cultural resonance, barriers to participation, and hopes for future programming (e.g., “What stood out most to you about the program?” “How—if at all—did culture, language, or ceremony influence your experience?” “What changes did you notice in your daily life?” “What should we keep, change, or add if the program continues?”). Responses were collected during the final gathering and documented in a de-identified format. We analyzed the reflections using an applied thematic approach suitable for community-engaged program evaluation. Two members of the research team reviewed all responses, generated initial codes, and iteratively grouped codes into higher-order themes. Themes were refined through discussion to ensure they reflected the language and priorities expressed by participants and to maintain fidelity to community meanings. Representative excerpts are presented below to illustrate each theme.

Importantly, the present intervention was not the research team’s first mindfulness program developed with this Tribal community. The pilot emerged from several years of community-engaged programming that included multi-week mindfulness courses, weekend retreats, intergenerational mindfulness activities incorporating cultural arts and shared food, and programs integrating mindfulness with movement, yoga, and dance. Community members and cultural leaders participated in the development and delivery of these activities across this period, allowing the approach to be iteratively refined before the present IndigenousMIND/MB-BP pilot. Thus, the high level of acceptability observed in the present study should be understood in the context of a longstanding community partnership and an intervention-development process that had already incorporated substantial community guidance regarding culturally appropriate content, format, and delivery.

## Results

### Qualitative themes from the final gathering

RTA of participant reflections collected at the closing community gathering identified five primary themes: (1) Connection and Community; (2) Cultural Identity and Preservation; (3) Mindfulness and Personal Transformation; (4) Intergenerational Healing and Responsibility; and (5) Challenges and Resilience. Across themes, participants emphasized the importance of a relational, judgment-free learning environment; the centrality of culture and language as pathways into practice; and the ways mindfulness was experienced as both personal regulation and collective responsibility.

### Theme 1: connection and community

Participants consistently described the group as a supportive relational container—one that held the realities of everyday stress while offering consistency, warmth, and a sense of belonging within the tribal community. Several participants emphasized that the program’s value was not only the content of mindfulness training, but the social conditions that made the practice possible: flexibility, openness, and a shared commitment to showing up for one another.

*I enjoyed the flexibility and the openness of everyone here, because as we went through those eight weeks together, life was just coming at us.* (P01:00:07)

*And that love—it was all understanding and family, showing up for each other.* (P02:01:29)

*Even if we didn’t know each other well, there was that sense of connection.* (P02:01:29)

Participants also highlighted they experienced mutual learning and collective grounding, particularly through shared attention, gratitude, and an explicit commitment to nonjudgment and education.

*It’s been a great experience learning from you all.* (P03:04:53)

*This time with all of you has definitely reminded me of the power and peace that can come from where we bring our attention and focus—on gratitude.* (P04:11:16)

*What I thought was important was that it was a judgment-free zone. And it wasn’t just saying that—it was also educating.* (P05:23:40)

### Theme 2: cultural identity and preservation

Participants described the program as reinforcing cultural pride and strengthening motivation to preserve and transmit Tribal traditions, especially to youth. Cultural elements—language, story, and explicit affirmations of tribal identity—were frequently described as a defining feature of the intervention, and a source of meaning-making that extended beyond stress reduction. For many, the program helped to foster intergenerational solidarity while facilitating a deeper appreciation for practicing tribal traditions that had been disrupted through colonization.

*We are trying to hold onto those traditions and pass them down to the next generation and our youth.* (P02:01:29)

*We need to teach our children. We need to bring our tribe back together. We need to be who we are as (our nation)—you all brought that up.* (P06:29:51)

“As we’ve explored mindfulness, we see that at the heart of it is something religious and spiritual. That heart is inextricable from our mindfulness. It makes sense of everything else we do. Everything else falls apart unless it’s rooted in something deeply spiritual.” (P*:36:00)

Participants located this work within a longer arc of cultural continuity and survival, describing Tribal identity as enduring and actively preserved across generations.

*(Our nation) stands out and is well known for being a powerful tribe, preserving our culture for 350 years, and holding powwows.* (P07:15:07)

Language, in particular, was experienced as both emotionally resonant and practically engaging—something participants could feel in their bodies through song and participation.

*I loved hearing the language. I could even sing a little when you were singing. I enjoyed that so much.* (P08:22:08)

“You made it connect to us. I’ve had Peloton memberships, mom has had meditations before. This was geared to us and with our language… which made it so much more powerful. That was the best or the highlight for me.” (P*:05:01)

Several reflections suggested that mindfulness practices became a bridge into ancestral presence and place-based memory, linking contemplation with land, ocean, and elders’ histories.

*Listening to the stories about elders, after going to the classes, I sit and do my meditation…thinking about what my ancestors did in that place—by this ocean.* (P09:28:44)

### Theme 3: mindfulness and personal transformation

Participants described mindfulness as a practical and durable shift in daily life—often expressed through new habits of attention, sensory awareness, and time spent outdoors. For some, “listening” practices became a preferred and accessible pathway into meditation.

*Someone mentioned listening to the sounds around us. Now, almost every day, I go outside and sit for about 20 minutes. This has become my go-to meditation.* (P10:06:23)

Although blood pressure change could not be quantitatively evaluated in this pilot, one participant spontaneously described a perceived improvement in blood pressure during the qualitative discussion. This statement is presented as part of the participant’s experience and should not be interpreted as an objectively measured cardiovascular outcome.

I think the thing that I learned, that has stuck with me, was… someone had pointed out, that besides listening to our breath, that’s what I generally focus on, the ‘In-breath’ and the ‘Out-breath’, someone had mentioned listening to sounds. So now, almost every day, I go outside and sit for about 20 minutes. This has become my go-to meditation. I feel so fortunate… In my backyard there are trees and birds—so many birds. So many bird sounds, soft ones and loud ones, it really centers me to have that sound meditation. *I’ve found that my blood pressure has come down. It had been creeping up, and now it’s back down.* (P11:06:23)

Participants also described mindfulness as reshaping how they related to painful histories and present-day reminders of Indigenous land dispossession, emphasizing a deliberate practice of redirecting attention toward beauty, land, and what remains life-giving. For one participant, this shift in perspective fostered a deeper appreciation for the natural world, including plants as meaningful relations that served as enduring expressions and reminders of their culture.

*Instead of looking at the houses and thinking, ‘These people have stolen this land,’ I bring myself to that space of beauty on either side of the road. Don’t look at the houses—look at the trees*. (P12:25:07)

Importantly, mindfulness was repeatedly reframed as an existing ancestral practice that was disrupted through the process of colonization. Participants rejected the idea that mindfulness is only “Eastern,” describing Indigenous contemplative practices as longstanding and embedded in song, dance, prayer, and observation of the natural world.

*Dance is mindfulness. Song is mindfulness. All these forms of prayer and ceremony lie at the heart of the practice because they bring us to that oneness*. (P13:37:27)

*Mindfulness is really part of who we are. When we talk about mindfulness, it often seems very Eastern. But our people sat outside and observed the beauty we live among—if we open our eyes to see it.* (P12:25:07)

### Theme 4: intergenerational healing and responsibility

Participants emphasized that the program’s value extended beyond individual stress reduction toward collective responsibility—reviving community practices, strengthening youth engagement, and restoring intergenerational transmission. Talking circles and other collective traditions were described as necessary pathways for returning to “who we are.”

*We need to get back to having talking circles. We need to get back to incorporating this—because this is who we are.* (P07:15:07)

Several reflections explicitly centered youth involvement and cultural continuity as central aspirations for future iterations of the program.

*The younger generation will get together and pass all of this on. I’ll be at the next class.* (P14:21:59)

*I hope we can get our young people involved, because so many of them are moving into mainstream society and away from their culture.* (P15:14:39)

Participants also highlighted perseverance, self-encouragement, and recognition of each person’s contribution—suggesting the program supported both personal agency and collective esteem. Through this lens, every generation had inherent gifts that were necessary for community wellness.

*I tell myself, don’t give up. Do what you can. My gift is different from everyone else’s. And everyone else has a gift here.* (P16:20:50)

Reflections also extended into family contexts, with mindfulness prompting participants to consider how stress and coping play out in close relationships and across generations. This intergenerational healing offered skills of self regulation and fostered empathy for those of differing ages within their family unit.

*Listening to the stories about elders made me reflect on my parents. My father is hot-headed. When the doctor tells him to sit down and relax, he doesn’t listen* (P17:27:40)

### Theme 5: challenges and resilience

Alongside benefits, participants spoke candidly about social stressors—bullying, exclusion, and interpersonal strain—and the emotional difficulty of maintaining openness in spaces that do not always feel welcoming. These reflections underscored that mindfulness practice occurred within real social conditions, not apart from them.

*It’s not right that people are out here bullying others because they think they’re better than the next person* (P18:30:25).

*But sometimes it’s hard. Sometimes it’s scary. It doesn’t feel good if it doesn’t feel welcome by everyone* (P19:20:07).

Participants also described resilient responses that balanced care, boundaries, and commitment to personal integrity.

*I love my people, even when they’re mean. Sometimes I give it right back with the same amount of care. Otherwise, I just feel sorry for them* (P20:31:14).

*Don’t dim your light because it’s too bright. I’m mindful about that* (P21:19:24).

Although participants did not identify specific changes to the cultural content or overall structure of the intervention in the formal qualitative reflections, informal comments to the research team included suggestions from some participants that individual sessions could be shorter. Because these comments occurred outside the formal qualitative data collection process, they were not included in the thematic analysis. Nevertheless, this feedback was documented as an implementation consideration for future iterations of the program.

## Discussion

This study documents the development and early implementation of IndigenousMIND/MB-BP, an eight-week, culturally grounded hypertension-prevention and wellness program created by integrating the IndigenousMIND intervention development approach with the Mindfulness-Based Blood Pressure Reduction (MB-BP) curriculum. The primary contribution of this work is not only evidence of feasibility and acceptability in a Tribal community setting, but also the creation of a structured, portable protocol that other Indigenous communities can adapt for their *own* health priorities and cultural contexts.

At a broad level, IndigenousMIND/MB-BP responds to a persistent gap in cardiovascular health inequity efforts: while decades of research indicate that culturally grounded programming, land/place connection, and collective approaches to wellness support psychosocial health in Indigenous communities, fewer programs provide replicable implementation guidance that communities can sustain beyond a pilot period. This gap is especially consequential for hypertension, given its central role in cardiovascular morbidity and mortality and the established population-level benefits of even modest reductions in systolic blood pressure (41). The challenge is not simply identifying “what works,” but building delivery models that communities recognize as theirs—ethically governed, culturally resonant, and operationally feasible.

Participants’ reflections underscore a central premise of IndigenousMIND: for many Indigenous communities, cultural and spiritual continuity is not separate from health behavior change—it is a pathway into it. Themes around cultural identity, language, and ancestral place-based reflection suggested that participants experienced the program as a form of reconnection rather than merely a skills class. This matters because hypertension interventions often require sustained behavior change in domains strongly shaped by stress physiology, environmental context, and lived experiences of marginalization. Historical trauma frameworks emphasize that colonization can produce enduring multilevel harms—individual, family, and community—through pathways that include ongoing stress exposure, disrupted cultural continuity, and constrained access to safe, affirming health systems (18, 42). In this light, culturally grounded intervention design can be understood not only as “tailoring for acceptability” but as addressing core determinants that shape the feasibility of long-term self-regulation and health behavior maintenance within Indigenous communities.

Participant statements that “dance is mindfulness” and that mindfulness “is part of who we are” indicate that IndigenousMIND/MB-BP functioned, in part, by recognizing mindfulness as a relational Indigenous practice rather than an imported modality. This is consistent with broader calls to attend to cross-cultural meaning-making and the risks of decontextualizing contemplative practice when delivering mindfulness in diverse settings (43). Importantly, the Tribal-specific integration of language and culturally meaningful metaphors appeared to activate pride, belonging, and motivation—mechanisms that may support adherence to practices required for sustained blood pressure benefits.

MB-BP is grounded in a mechanistic model in which mindfulness training strengthens self-regulation (attention control, emotion regulation, interoceptive awareness) and thereby supports healthier choices relevant to blood pressure (e.g., stress reactivity, diet, physical activity, medication adherence). Clinical trials demonstrate clinically meaningful reductions in systolic blood pressure, improvements in diet-related outcomes, reductions in sedentary behavior, and even reductions in depression symptoms (31, 34, 44). IndigenousMIND, by contrast, is explicitly designed to position Indigenous culture as medicine—embedding ceremony, language, and community-defined practices within mindfulness-based structure so that health behavior change is nested inside cultural revitalization rather than treated as a separate “lifestyle” agenda.

IndigenousMIND/MB-BP is therefore best conceptualized as a “function-preserving” integration: it retains MB-BP’s evidence-based behavior-change scaffolding while strengthening cultural resonance and relational accountability through IndigenousMIND’s adaptation logic. Participant feedback from this pilot aligns with that premise. Reflections emphasized (a) mindfulness practice adoption (e.g., daily outdoor listening), (b) shifts in cognitive appraisal and emotion regulation (e.g., redirecting attention toward beauty and land), and (c) strong group cohesion and nonjudgment—factors plausibly supportive of sustained engagement with MB-BP-aligned home practices and cardiovascular risk-reduction behaviors.

A major output of this project is the IndigenousMIND/MB-BP protocol that can be used as a practical dissemination product (see Appendix A). In many culturally grounded interventions, adaptation occurs through specific skillsets and experiences held by facilitators, making replication difficult even when results are promising. Here, the intervention was created through a structured approach: observe an MB-BP session, debrief and map the “active ingredients,” adapt with cultural content, deliver within the community, and document the final form in a protocol and handbook. This process directly addresses widely recognized needs in dissemination and implementation science: adaptations will happen, and the mindfulness field benefits when they are transparent, theory-linked, and documented for future implementers (37, 38).

The protocol is intended to function as (1) a culturally grounded implementation guide, (2) a fidelity-support tool that clarifies session intent and core functions, and (3) an adaptation framework with prompts and examples that Tribal teams can use to integrate their own language, stories, and practices. Other Indigenous communities may use the protocol as a starting point for locally led co-design rather than as a culturally universal intervention. This approach may support scalability while respecting Tribal sovereignty and place-based difference. Importantly, this culturally responsive protocol responds to recent calls for repowering (45) in public health, extending this principle to mindfulness research by acknowledging the enduring impacts of colonialism while supporting Tribal communities in reclaiming and leveraging their own knowledge systems and cultural practices to advance place-based, culturally grounded health and well-being.

The introduction for this paper emphasizes that colonization and structural racism can erode cultural strengths and create conditions in which standard interventions feel externally imposed or misaligned with Indigenous community practices and values. We wanted to know if we could develop an appropriate mindfulness program as a way to explicitly reverse those trends. The qualitative themes provide coherence with that narrative in three ways. First, the prominence of connection/community suggests that social and relational mechanisms were central to participants’ experience. This aligns with CBPR and collective healing frameworks that highlight how interventions can function as community infrastructure rather than solely individual change programs (39). In practical terms, it suggests that group climate and relational facilitation are not “soft” implementation details but core components that may influence engagement and adherence.

Second, cultural identity/preservation themes support the notion that cultural continuity is itself experienced as health-promoting. Participants explicitly linked the course to teaching youth, preserving language, and being “who we are as (our Tribe).” This suggests the intervention may have worked partly by strengthening identity and meaning—pathways that historical trauma scholarship frames as protective and reparative (18, 42).

Third, mindfulness/personal transformation themes were often expressed through ecological attention and place-based language (e.g., listening outdoors; attending to trees rather than houses symbolizing dispossession). This points to a distinctive “ecological mindfulness” mechanism, in which environment and land are not background context but active supports for regulation and meaning-making. Such mechanisms may be particularly relevant in Indigenous wellness frameworks that emphasize balance among mind, body, spirit, emotion, and relationship to creation (20).

Although overall acceptability was high, informal feedback that some participants would prefer shorter sessions suggests that session duration should be examined as an implementation consideration in future iterations. Future work should assess whether modifications to session length can reduce participation burden while preserving the intervention’s core functions and opportunities for cultural and relational engagement.

This pilot was not designed to evaluate blood pressure efficacy, and blood pressure data were not sufficiently complete to permit a quantitative pre-post analysis. Although blood pressure measurement was initially incorporated as an exploratory component of the pilot, obtaining consistent participant-reported measurements proved difficult and collection was discontinued. This experience represents an important feasibility finding for subsequent evaluation of IndigenousMIND/MB-BP: future efficacy studies will require standardized investigator-obtained blood pressure measurement procedures and an adequately powered sample selected for elevated blood pressure. Moreover, participants in the present protocol-development study were not required to have hypertension or elevated blood pressure, further limiting the appropriateness of evaluating blood pressure reduction as an outcome at this stage.

The absence of blood pressure efficacy data in the present study should therefore be considered in relation to its developmental purpose. The cardiovascular intervention being culturally adapted—MB-BP—has previously been evaluated in larger clinical studies specifically enrolling participants with elevated blood pressure, with randomized trial findings demonstrating clinically meaningful reductions in systolic blood pressure. Thus, the present study addresses a different but complementary question: whether the evidence-based functions of MB-BP can be integrated into a culturally grounded, community-engaged Indigenous intervention while maintaining acceptability and producing a structured protocol suitable for subsequent efficacy testing. A logical next stage is therefore an adequately powered trial recruiting participants with elevated blood pressure and prospectively evaluating whether IndigenousMIND/MB-BP produces clinically meaningful changes in blood pressure and associated cardiovascular risk factors.

Several limitations of this pilot should be considered. The small, community-based convenience sample was recruited for intervention and protocol development and was not intended to be statistically representative of the broader Tribal population. In addition, incomplete participant-provided blood pressure measurements prevented quantitative evaluation of pre-post blood pressure change, and the study was not designed or powered to evaluate cardiovascular efficacy. Although overall acceptability was high, informal feedback regarding session duration suggests that participation burden and optimal session length warrant further examination. Finally, IndigenousMIND/MB-BP was developed in partnership with a specific Tribal community and should not be interpreted as a culturally universal intervention for Indigenous Peoples. Implementation in other Indigenous communities will require locally led adaptation incorporating each community’s language, cultural practices, priorities, governance, and understandings of health and healing. These limitations inform the next phase of research, including more systematic assessment of engagement, standardized cardiovascular measurement, and evaluation in a larger sample selected for elevated blood pressure.

From a health equity perspective, the goal is not only “effectiveness” in a controlled trial but durable community-owned implementation. IndigenousMIND/MB-BP is explicitly oriented toward sustainability: the protocol and handbook aim to support community delivery capacity and reduce dependence on external experts. This orientation may be especially important given the documented barriers Indigenous communities face in accessing culturally safe care and the mistrust that can result from experiences of discrimination in healthcare settings (46). While clinical systems have critical roles, community-embedded interventions may offer parallel pathways to cardiovascular prevention that feel safer and more culturally congruent.
