# Supplementary material for: Designing a culturally responsive mindfulness-based blood pressure program: the IndigenousMIND/MB-BP protocol
Source: Front Public Health. 2026 Oct 21;14:1911281. doi: 10.3389/fpubh.2026.1911281 (PMC13731262; doi:10.3389/fpubh.2026.1911281)
Supplement: Supplementary file 1 [file Data_Sheet_1.PDF]

Supplemental Protocol:
IndigenousMIND/ Mindfulness-Based Blood Pressure Reduction Program

This supplemental protocol provides the week-by-week structure used in the Narragansett IndigenousMIND/Mindfulness-Based Blood Pressure Reduction (IM/MB-BP) blood pressure reduction program. It is intended to accompany the manuscript and should be read in close connection with the full description of the intervention development process, community engagement procedures, and cultural adaptation framework. The protocol is offered as an example of how Mindfulness-Based Blood Pressure reduction (MB-BP) practices can be integrated with Indigenous cultural teachings, language, ceremony, movement, and community-based reflection. It is not intended to be used as a stand-alone manual by individuals without substantial preparation.

Implementation of this program should be led by facilitators with formal mindfulness teacher training, an established personal mindfulness practice, and prior experience teaching mindfulness-based programs and/or mindful movement. Formal mindfulness teacher preparation typically requires multiple training periods, supervised teaching, retreat practice, mentorship, and years of personal practice; it should not be understood as something that can be developed quickly through a short workshop. Ideally, facilitators should already have experience teaching mindfulness, meditation, and mindful movement or yoga, and should work closely with Indigenous cultural leaders, language keepers, elders, community members, and local advisors to determine how cultural practices should be respectfully embedded in the curriculum.

The protocol below illustrates one way that cultural inflection points were incorporated into an 8-week curriculum developed in collaboration with a specific Indigenous Nation in the Northeastern United States. These adaptations included practices such as smudging, dedication, Narragansett language, talking circles, cultural movement, breath practices, and community-guided reflection. The practices included here are specific to the cultural and relational context in which the program was developed and should not be copied uncritically into other communities or Tribal Nations.

In several places throughout the protocol, specific cultural words, phrases, or practices from the collaborating Tribal Nation have been intentionally redacted. However, the placement of these elements has been preserved to illustrate how other Indigenous communities may thoughtfully incorporate their own language, teachings, ceremonies, movement traditions, ecological relationships, or cultural concepts into the curriculum in ways that are locally meaningful and community-led.

Communities interested in adapting this program are encouraged to work closely with local elders, cultural leaders, language keepers, and community members to determine which practices, teachings, arts, movement forms, relational structures, or land-based traditions are appropriate for their own setting. Groups may also benefit from consultation with formal mindfulness teacher training programs and Indigenous-led organizations, such as the Indigenous

Mindfulness Coalition, to support the development of culturally grounded teaching teams, ethical adaptation processes, and community-engaged implementation.

IM/MB-BP IS NOT INTENDED TO BE, AND SHOULD NOT BE USED AS, MEDICAL OR PROFESSIONAL ADVICE OR A SUBSTITUTE FOR MEDICAL TREATMENT BY A HEALTHCARE PROFESSIONAL, SUCH AS A CARDIOLOGIST OR PRIMARY HEALTHCARE PROVIDER. Rather, IM/MB-BP is designed to be complementary and integrative with evidence-based medical care. For this reason, IM/MB-BP is best delivered under the consultation of, and partnership with, your healthcare provider. While clinical trials have shown evidence that MB-BP reduces blood pressure on average, individual results are not guaranteed. You should consult with a healthcare provider to see if IM/MB-BP may be right for you. Brown University is in the process of developing a teacher training program for MB-BP. Those interested are encouraged to enroll in it to become certified MB-BP teachers yet adapt MB-BP to the IM/MB-BP and local tribal context.

IM/MB-BP is not provided during a professional relationship between a healthcare provider and a patient. IM/MB-BP is not recommending a course of treatment for your circumstances or making a diagnosis using IM/MB-BP. Do not disregard or delay in seeking medical advice or treatment because of any information you receive through IM/MB-BP. Your healthcare provider should be consulted regarding matters concerning medical conditions, treatment, and needs of you and your family.

Orientation

1. Introduce yourself and welcome everyone.
    - State experience in teaching mindfulness and/or Indigenous contemplative practices.
    - State knowledge/training in hypertension etiology and/or treatment.
    - State experience connecting mindfulness, Indigenous wellness approaches, and hypertension risk factors (e.g., diet, physical activity, weight loss/maintenance, medication adherence).
2. Give an outline of the whole orientation.
    - Give a brief history of MBSR, IndigenousMIND, and IM/MB-BP.
    - Discuss IM/MB-BP as complementary to medical treatment.
    - Review evidence supporting IM/MB-BP.
    - Highlight mindfulness research, Indigenous wellness perspectives, and IM/MB-BP findings.
3. Introduce mindfulness and Indigenous mindfulness.
    - Mindfulness is paying attention, on purpose, in the present moment, nonjudgmentally.
    - Discuss how many Indigenous cultural practices naturally cultivate present-moment awareness through relationship with community, movement, storytelling, language, nature, ceremony, and creativity.
    - Cultivate curiosity, exploration, kindness, and friendliness toward experience.

- Introduce mindfulness as remembering (*sati* in Pali), including remembering to bring wisdom into the present moment.
- Discuss applying wisdom related to lowering blood pressure and fostering health.
- Discuss noticing when the mind moves to the past or future and returning to present-moment awareness.

4. Guided mindfulness and grounding practice.
    - Introduce posture options (chair, cushion, standing).
    - Focused attention meditation using breath, body, palms, soles of feet, sound, or connection to the natural environment as anchors.
    - Introduce awareness practices grounded in Narragansett cultural teachings, movement, language, or relationship with the Earth.
    - Open awareness of thoughts, emotions, and physical sensations.
    - Return attention gently and nonjudgmentally to the anchor.
5. Group introductions.
    - Discuss confidentiality and respectful listening.
    - Explain that confidentiality includes refraining from discussing others' sharing outside of class.
    - Discuss no recording or photographs without permission.
    - Participants introduce themselves and may comment on the meditation or cultural practices discussed.
6. Health reflection activity.
    - Review health feedback or self-reflection regarding blood pressure and health behaviors.
    - Invite participants to notice thoughts, emotions, and bodily sensations arising during reflection.
7. Introduction to hypertension and cardiovascular disease.
    - Describe modifiable hypertension risk factors including obesity, diet, salt intake, physical activity, stress, and medication adherence.
    - Discuss environmental and social contributors to hypertension and craving.
    - Discuss mindfulness and Indigenous contemplative practices as supporting awareness, self-regulation, and relationship to health behaviors.
    - Emphasize individualized pathways and areas of focus.
8. Logistics of the class.
    - Review class schedule and all-day retreat.
    - Review course structure including meditation, cultural movement practices, body scan, walking meditation, mindful eating, talking circles, and community reflection.
    - Review home practice expectations and online learning materials if applicable.
9. Self-care guidance.
    - Encourage participants to modify movement and posture as needed.

- Encourage participants to rest or adapt practice if overwhelmed physically or emotionally.
- Encourage comfortable clothing and bringing needed supports.

10. Attendance and home practice expectations.
    - Emphasize home practice as integral to the program.
    - Discuss challenges of making time for practice.
    - Emphasize attendance and arriving on time.
11. Risks and benefits discussion.
    - Physical risks related to movement practices.
    - Emotional risks related to awareness of difficult emotions or trauma.
    - Relational changes that may occur through mindfulness and self-reflection practices.
    - Time commitment involved in practice.
    - Potential benefits including increased awareness, improved well-being, enhanced self-care, and greater community and cultural connection.
12. Review medical disclaimer.
13. Individual reflection.
    - What intention do I bring into this program?
14. Talking Circle.
15. Closing meditation, grounding, or cultural reflection practice.

Week 1: The Strength Within

1. Read Week 1 class theme: "The Strength Within."
2. Emphasize the theme: There is more right with you than wrong.
3. Opening smudge and dedication in Narragansett.
4. Opening meditation: "Olfactory Sense," adapted from Dr. Michael Yellow Bird's shared meditation.
5. Review guidelines for participation, including confidentiality and self-care.
6. Guided individual reflection: What has brought you here? What do you hope to achieve?
7. Talking Circle.
    - Participants may use the smudging feather as an anchor while sharing.
    - Participants are invited to use the breath as an anchor while listening, remembering to breathe in and breathe out as others speak.
8. Body movement stretch: *Indigenous word*, meaning "stretch out" or "to give out from."
    - Mountain pose: *Indigenous word*.

- Crescent Moon: *Indigenous word*.
- Spinal twist: *Indigenous word*
- Forward bend: *Indigenous word*
- Backbend with arms overhead: *Indigenous word*
- Mountain pose: *Indigenous word*

9. Reading from Thich Nhat Hanh, "Breathing to Quiet the Mind and Body to Realize Joy."
10. Mindful eating exercise using a raisin, blueberry, grape tomato, or similar food.
11. Discussion of adiposity and awareness of personal triggers.
12. Body scan practice, emphasizing that participants can always return to the anchor point: *Indigenous word*, the breath.
13. Talking Circle focused on participants' experiences with the body scan.
14. Home practice assignment.

Week 2: Mindfulness, Food, and the Wandering Mind

1. Read Week 2 class theme.
2. Opening smudge and dedication in local language.
3. Opening meditation and brief body scan.
4. Talking Circle to discuss home body scan practice.
5. Review guidelines for participation, including confidentiality and self-care.
6. Discuss the wandering mind and the purpose of cultivating stillness.
7. Close this portion with a Washing Prayer.
8. Brief mindful movement practice, including standing mountain pose.
9. Five-minute break.
10. Talking Circle.
    - Discuss the experience of using mindfulness while eating a meal.
    - Reflect on one's relationship with food.
    - Participants may use the smudging feather and/or breath as an anchor while speaking and listening.
    - Describe key modifiable hypertension risk factors, including obesity, diet, salt intake, physical activity, and antihypertension medication adherence
11. Challenging food activity.
12. Nine Dots exercise and check-in with how the body feels.
13. Body movement stretch:(Insert Indigenous word here).
    - Mountain pose: (Insert Indigenous word here).
    - Crescent Moon: (Insert Indigenous word here).

- Spinal twist: (Insert Indigenous word here).
- Forward bend: (Insert Indigenous word here).
- Backbend with arms overhead: (Insert Indigenous word here)..
- Balance pose.
- Mountain pose: (Insert Indigenous word here).

14. (Insert Indigenous word here; what is the object focused on?) for focused meditation.
15. Medicine Wheel reflection.
16. Home practice assignment.

Week 3: Movement, Pleasant Events, and Embodied Awareness

1. Opening and arrival practice.
2. Formal mindfulness practice.
    - Long and loose.
    - Relaxed and alert.
    - Comfortable.
3. Group discussion.

    Discuss how physical activity or diet affect hypertension risk factors, potential discussion around obesity. What are barriers to accomplishing goals?
4. Walking meditation with intentional stepping (if possible, use as many Indigenous words as possible, including words for walking, or for the statements below).
    - “I have arrived.”
    - “I am home.”
    - “Home in the here and now.”
    - Option to blend walking meditation with dance or culturally meaningful movement.
5. Transition to floor meditation.
6. More strenuous movement reflection.
    - Envision your preferred physical activity, such as walking, swimming, gardening, dancing, or housework.
        - Ask: What is your preferred way to move your body?
    - Reflect on how the body feels before, during, and after a preferred form of movement.
    - Discuss how Indigenous people have envisioned the body and health.
7. Pleasant Events Calendar practice.
    - Bring to mind a pleasant event from today or long ago.
    - Notice the environment, who was present, and what physical sensations were present.
    - Notice emotional sensations.

- Notice thoughts that were present at the time.
- Return to the anchor point.
- Notice present-moment feelings, emotions, thoughts, gratitude, joy, or a sense of being fortunate.

8. Home practice assignment for Week 3.
9. Closing guided meditation.
    - Review the evening's practices.
    - Review home practice and preview the next class.
    - Close with the poem "Enough."

Week 4: Stress, Self-Awareness, Movement, and SMART Goals

1. Opening.
2. Review group reminders.
    - Mute when not speaking.
    - Follow Zoom etiquette if meeting remotely.
    - Avoid cross-talking.
    - Stay on topic.
    - Allow time for all participants to share.
3. Discuss lowering stress through focused attention and self-awareness.
4. Meditation with an anchor point: breath, body part, or sound, for approximately 10 minutes.
5. Unpleasant Events Calendar.
    - Each participant writes about an unpleasant event experienced during the week.
6. Whiteboard exercise on responses to stressors.
    - Identify physical sensations, emotions, feelings, and thoughts.
    - Use arrows to show how stress responses unfold.
    - Introduce the possibility of offering kindness to oneself.
7. Kindness meditation for approximately 10 minutes (how is "kindness" reflected in local culture?).
8. Physical movement for approximately 25 minutes.
    - Emphasize raising the pulse through accessible activity.
    - Participants notice physical sensations, emotions, and thoughts during movement.
    - Examples may include walking faster than usual, pickleball, running, basketball, hula hooping, or other appropriate activities.
9. Break and return.
10. Stretching poses, using Indigenous-led video or another community-approved movement resource.

11. Talking Circle focused on experiences during and after physical movement.
12. Week 4 home practice: set an intention or SMART (Specific, Measurable, Attainable, Relevant to lowering blood pressure, and Time-bound) goal for the week related to a determinant of blood pressure you would like to improve, such as:
    - Diet.
    - Weight loss.
    - Physical activity.
    - Reduced alcohol consumption.
    - Blood pressure medication adherence.
13. Discuss examples of SMART goals.
14. Closing meditation focused on how the body feels after movement has stopped.

Week 5: Attention Control, Open Monitoring, STOP Practice, and New Paths

1. Object meditation to reduce stress.
    - Participants select an object of attention, such as the breath, body sensations, or sound. Use the Indigenous words for breath, body, or sound and a story that applies to those words.
    - When the mind wanders, participants gently bring attention back.
    - Participants notice physical sensations where the body touches the chair, floor, or other surface.
    - Participants observe whether sensations shift moment to moment or remain steady.
    - Participants shift awareness to emotional sensations such as joy, irritation, or neutrality.
    - Participants notice whether emotions feel generally positive, negative, or neutral.
    - Participants shift awareness to thoughts and observe them with kindness and curiosity.
    - Participants listen to the soundscape as a way of strengthening attention control and present-moment awareness.
2. Open monitoring/sitting meditation.
    - Participants notice smell, taste, touch, sound, physical sensations, emotions, and thoughts.
    - Participants observe lived experience as it is in the present moment.
    - Participants compare what is present now with what was present at the beginning of practice.
    - Participants return to the object meditation, such as breath, body part, or sound.
3. Guided midpoint reflection.
    - The program is about halfway complete.
    - Reflection questions:
        - What am I learning?
        - How does it show up in my life?

        - How am I engaging with weekly practice?
        - Am I willing to commit to the next half of the class?
        - Am I willing to be fully engaged?

4. Breakout rooms for participants to share reflections.
5. Mindful movement or 25-minute walk.
6. Whole-group check-in.
    - Share how participants respond to stressors.
    - Become aware of thoughts, emotions, and sensations.
    - Practice pausing in order to respond more skillfully rather than reacting automatically.
7. STOP practice.
    - S: Stop.
    - T: Take a breath; take a moment.
    - O: Observe.
    - P: Proceed.
8. Discuss taking a new path.
    - When stressors arise, participants are invited to take a new path rather than follow old habitual patterns.
    - Example: Instead of eating a pint of ice cream in response to stress, one might take a walk or have a cup of tea.
    - Participants use attention control strategies to support new paths.
9. Intentions or SMART goals.
    - Discuss how the previous week went.
    - Emphasize the importance of kindness toward yourself throughout the behavior change process.
    - Revisit setting an intention or SMART goal for the coming week to improve a determinant of blood pressure that you would like to change. This may be the same as, or similar to, last week's intention, or it may be a new goal related to diet, alcohol reduction, physical activity, weight management, or adherence to blood pressure medications.
10. Home practice assignment.
    - Introduce the open monitoring/sitting meditation recording.
    - Emphasize that the recording includes more silence to support deeper independent practice between guided instructions.

Week 6: Open Monitoring, Four Directions, Interpersonal Mindfulness, and STOP

1. Sitting/open monitoring meditation: Use Indigenous words for object of meditation.

2. Opening.
3. Practice on bringing ourselves back to peace, including Four Directions reflection or practice.
4. Yoga or mindful movement.
5. Talking Circle focused on how participants felt during meditation and how their intentions or SMART goals are unfolding.
6. Discussion of interpersonal mindfulness.
   - Use chat or breakout rooms if meeting remotely.
7. Review STOP strategy.
   - Stop.
   - Take a moment.
   - Observe.
   - Proceed.
8. Closing sitting/open monitoring meditation.

Week 7: Self-Directed Practice, Perspective, and Nourishing versus Draining Patterns

1. Sitting/open monitoring meditation: Use Indigenous words for object of meditation.
2. Introduction.
   - Explain that the class will shift to a more personal level.
   - Ask: What will help you the most?
   - Ask: What might we want to offer ourselves?
3. Ten minutes of self-directed mindful movement.
   - Participants offer themselves a set of mindful movements.
   - Participants ask: What is the body asking for right now?
   - Participants explore how it feels to practice in community without direct guidance.
   - Participants compare how the body feels before and after mindful movement.
   - Participants are invited to offer themselves kindness and support.
4. Community reminder.
   - Mute devices when not speaking.
   - (if online) Keep cameras on if possible to support a sense of community.
5. Seated reflection and perspective practice.
   - Participants close their eyes and notice the sensations, emotions, and thoughts present in the seat they are sitting in. If possible, do this outdoors and focus on how ancestors have made teachings about relationships to the Earth and use that perspective to guide how people engage in this exercise.
   - Participants open their eyes and look around the room.

- Participants move to a new location and notice how it feels.
- Reflection questions:
    - Is there a new perspective?
    - Do I tend to stay in similar places?
    - Do I want to explore new places?
    - Can I be at home wherever I am?
    - Are there attachments to certain places?
    - Where am I in my life?
    - Where am I at this moment?
    - Where am I going?

6. Ten-minute self-directed meditation.
    - Participants may stay in the new seat or return to their original seat.
    - Participants choose one practice:
        - Focused attention using breath, body part, or sound.
        - Open monitoring or sitting meditation (using Indigenous words for objects of focus).
        - Walking meditation (emphasize words for walking or observing).
        - Yoga or mindful movement (focus on the Indigenous yoga teaching from earlier).
        - Body scan (use local language for the body parts).
        - Loving-kindness meditation (use poetry or other teaching from the tribe to support this meditation).
        - Visual meditation using an object in the visual field.
    - Facilitator gives a one-minute warning before the practice ends.
    - Participants are reminded that only one class remains and that the goal is to use meditation to support heart health, reduce anxiety, and lower blood pressure.
7. Talking Circle.
    - Discuss the experience of self-directed practice.
    - Reflect on the past week.
    - Revisit intentions or SMART goals.
    - How does this work meet the emphasis on health from an Indigenous perspective?
8. Discussion of nourishing ideas versus draining ideas.
9. Mountain (use Indigenous word for "mountain and include same language in describing objects on the mountain) meditation.

10. Review Week 7 home practice.

Week 8: Integration, Future Self, and Carrying the Practice Forward

1. Opening.
2. Self-directed body scan.
3. Standing yoga.
   - Begin with a short body scan while standing in mountain pose (use local language to identify body parts; use Indigenous concept of ‘mountain.”)
   - Participants add whatever movement or posture they feel they need.
4. Ten-minute anchor meditation.
   - Participants use breath, body, or sound as an anchor (focus on Indigenous words for objects of focus; add a story if possible).
   - Shift into open monitoring or sitting meditation.
   - Notice physical sensations, touch, taste, emotions, and thoughts (add in Indigenous concepts of sensations, touch, etc.).
   - Notice whether experiences shift moment to moment or remain steady.
   - Return to the narrower focus of the anchor meditation.
5. Guided reflection.
   - What do I want to remember from this program? How is my effort affect the tribe or family?
   - Participants may set two or three short-term goals or intentions for the next three months.
   - Participants may also set two or three long-term goals or intentions for the next one to two years.
   - Reflect on how to work with difficulties that might arise.
6. Letter to future self.
   - Participants write a letter to their future self.
   - Prompt: What from this program would you like to share with your future self?
7. Breakout room discussion.
   - Discuss how the program was experienced, including whether it has been helpful or not. What are the implications for the wider community, if there are any?
8. Whole-group discussion.
   - Reflect on the past week.
   - Discuss nourishing versus non-nourishing experiences.
   - Consider whether and how participants will carry the practices forward.
   - Discuss use of a gratitude journal to record nourishing experiences.
   - Reflect on being present in interpersonal interactions.
   - Ask: What do you want to carry forward?

9. Review key program themes.
    - Creating an environment conducive to hypertension reduction.
    - Supporting self-regulation as a pathway toward lower blood pressure.
    - Building awareness of thoughts, emotions, and sensations.
    - Developing a more considered response rather than an automatic reaction.
    - Using STOP practice.
    - Creating new paths rather than relying on habitual responses.
10. Biofeedback and 8-week IM/MB-BP self-reflection activity.
11. Talking Circle.
    - How has the program helped you, if it has?
12. Closing meditation.
